# Vertebrate conserved non coding DNA regions have a high persistence length and a short persistence time

**DOI:** 10.1186/1471-2164-8-398

**Published:** 2007-10-31

**Authors:** Dorota Retelska, Emmanuel Beaudoing, Cédric Notredame, C Victor Jongeneel, Philipp Bucher

**Affiliations:** 1 Computational Cancer Genomics Group, Swiss Institute of Bioinformatics, Lausanne, Switzerland; 2 Office of Information Technology, Ludwig Institute for Cancer Research, Lausanne Branch, Lausanne, Switzerland; 3 Computational Cancer Genomics Group, Swiss Institute for Experimental Cancer Research, Lausanne, Switzerland; 4 Structural and Genetic Information, Centre National de Recherche Scientifique, Marseille, France

## Abstract

**Background:**

The comparison of complete genomes has revealed surprisingly large numbers of conserved non-protein-coding (CNC) DNA regions. However, the biological function of CNC remains elusive. CNC differ in two aspects from conserved protein-coding regions. They are not conserved across phylum boundaries, and they do not contain readily detectable sub-domains. Here we characterize the persistence length and time of CNC and conserved protein-coding regions in the vertebrate and insect lineages.

**Results:**

The persistence length is the length of a genome region over which a certain level of sequence identity is consistently maintained. The persistence time is the evolutionary period during which a conserved region evolves under the same selective constraints.

Our main findings are: (i) Insect genomes contain 1.60 times less conserved information than vertebrates; (ii) Vertebrate CNC have a higher persistence length than conserved coding regions or insect CNC; (iii) CNC have shorter persistence times as compared to conserved coding regions in both lineages.

**Conclusion:**

Higher persistence length of vertebrate CNC indicates that the conserved information in vertebrates and insects is organized in functional elements of different lengths. These findings might be related to the higher morphological complexity of vertebrates and give clues about the structure of active CNC elements.

Shorter persistence time might explain the previously puzzling observations of highly conserved CNC within each phylum, and of a lack of conservation between phyla. It suggests that CNC divergence might be a key factor in vertebrate evolution. Further evolutionary studies will help to relate individual CNC to specific developmental processes.

## Background

Large-scale conservation of non-coding genomic regions has been discovered by Dermitzakis et al, after alignment of the human chromosome 21 to homologous regions of the mouse genome. This work reported that protein-coding genes were more conserved overall than non-genic regions, thus giving a large-scale confirmation that evolutionary conservation is a hallmark of biological function. At the same time, it showed that numerous short non-coding DNA fragments were extremely highly conserved between human and mouse, but absent from the Drosophilids genome [1]. Subsequent work established that some of these sequences are highly conserved across all vertebrate species, whereas other are conserved only between pairs of species [2]. Regions of > 200 bp of perfect identity between human, mouse and rat have been called ultraconserved elements (UCE) [3]. Conserved non coding regions (CNC) are also referred to by others as conserved non-genic (CNG) regions[1], conserved non-coding elements (CNE) [4] or highly conserved elements (HCE) [5].

Although the conservation of these sequences pointed to an important biological role, their function remained elusive. A general confirmation of the functional relevance of CNC genomic sequences was given by Drake et al [6] who showed that the conservation is not due to lower regional mutation rate, but is best explained by purifying selection. In this study, a subset of conserved sequences shows SNP allele frequency shifts with magnitudes comparable to those for coding mis-sense variants, which suggests that they are likely to be under similar selective pressure.

In a recent work, Siepel et al [5] analyzed genomic conservation in multiple alignments from four different phyla: vertebrate, arthropods, nematodes, and fungi. They concluded that part of non coding bases are conserved in all genomes studied, but the fraction of conserved bases lying outside of exons of protein-coding genes is increasing with the complexity of the investigated lineage. Moreover, this study provided interesting clues about the function of non-coding sequences. In vertebrates, CNC regions are over-represented within 3'UTRs of regulatory genes, and show a strong enrichment in RNA secondary structure candidates. Non-coding RNAs are thus likely to contribute to the pool of CNC sequences [5]. However, non-coding RNAs of known function, as well as UTRs of protein coding genes, have diverse, and often moderate, degrees of human-mouse conservation [7]. These functional elements are thus more likely to contribute to the moderately conserved fraction of eukaryotic genomes than to the highly conserved fraction. Detailed studies on some of the highly conserved sequences demonstrated that some of them play important regulatory roles [8,3]; and recent large-scale study showed that a significant proportion of vertebrate highly conserved CNC have a tissue-specific enhancer function [9].

Here we present a comprehensive exploratory analysis of conserved sequence regions based on genome alignments. Our analyses are motivated by two main questions: (i) why are vertebrate CNC not conserved in insects, in contrast to coding regions, and (ii) does the evolution of non-coding DNA explain the apparently higher complexity of vertebrates (which is not due to an increased gene content, since the protein coding genes content of metazoan genomes is surprisingly similar). To address these questions we designed a measure to quantify the conserved genetic information between pairs of vertebrate and insect genomes, and proved that the proportion of non-coding bases in the conserved fraction is similar between these phyla.

We investigated the persistence length of CNC and conserved coding regions in the two lineages. Persistence length is the length of a genomic region over which a certain percentage of sequence identity is consistently maintained. The concept of persistence length is loosely inspired by physical models of polymers, and gives some indication about the internal organization of the conserved regions. For coding regions, it is probably related to the conserved ungapped blocks, which are readily recognizable in multiple alignments of distantly related proteins; it might also reflect the exon lengths distribution in vertebrates and insects. For CNC, we hypothesize that persistence length reflects the length of a functional unit of genetic information, and thus can give us insights into the function of these elements. The last part of our analysis focuses on the dynamic properties of conserved regions. Operationally, we determine persistence time as the evolutionary time interval after which sequence divergence appears to be accelerated or sequence similarity becomes undetectable. We established the evolutionary kinetics of CNC over time, and show that the persistence time differs strikingly between CNC and conserved coding sequences. These results explain and reconcile most previous observations about conservation of different genomic regions, and open the way to more detailed studies on kinetics of CNC evolution.

## Results

### Conserved Information

We analyzed Blastz pairwise whole genome alignments provided by the UCSC Genome Browser (UCSC Genome Center). We chose to systematically compare human-chicken alignments for vertebrates to *Drosophila melanogaster*-*D. virilis *alignments for drosophilids, since the unconstrained mutational distances are very close for these species pairs (Additional file 1), and all the pairs are distant enough to allow a clear separation between conserved and neutrally evolving genomic fractions. Functional annotation for the analyzed genomes was extracted from Ensembl. A preliminary analysis of the studied alignments is shown in Table 1. Most coding sequences are included in the alignments for all genome pairs. 69.45% of human CDS are included in human-chicken (Hs-Gg) alignments, 96.55 % of *D. melanogaster *CDS in *D. melanogaster*-*D. virilis *(Dm-Dv) alignments. Repeats are the least alignable sequence class for the Hs-Gg genome pair, whereas the proportion of repeats and non-coding sequence aligned for Dm-Dv is extremely similar.

**Table 1:** Proportion of bases covered by pairwise alignments

	***Hs-Gg***	***Dm-Dv***
**CDS**	69.45%	96.55%
**REP**	0.38%	65.33%
**NC**	4.21%	63.86%
**Total**	3.56%	70.03%

Human basepairs in each functional genomic class included in human-chicken alignments (Hs-Gg), and of *D. melanogaster *basepairs included in alignments with *D. virilis *(Dm-Dv).

For each functional sequence class (coding, non-coding, repeats), we assessed the distribution of sequence identity through measurable intervals (see Methods). The sequence identity of coding regions peaks at 70–80% for both Hs-Gg and Dm-Dv, (Additional file 2). We performed a similar analysis for a larger number of vertebrate and insect pairs. As expected, more closely related vertebrate species peaked at a higher percentage of identity (80%–90% for the *Homo sapiens *– *Mus musculus *alignments, 85%–95% for the *Homo sapiens *– *Canis familiaris *alignments, and 75%–85% for *D.melanogaster*-*D.pseudoobscura *alignments, not shown). We limited our analysis to two pairs having directly comparable coding sequence identity distribution. The observation that CDS identity is the same in Hs-Gg and Dm-Dv genome pairs is consistent with the values reported in the respective genome sequencing papers [10,11], and with neutral genomic distances between these 2 species (see Additional file 1). These values can be explained by a faster evolution of the Drosophilid species, confirmed by several independent measures of genomic distance [12,13].

We first computed the distribution of each sequence class in different sequence identity bins. Following Margulies et al [14], we consider the distribution of sequence identity in repeats as a measure of neutral divergence. The proportion of repeats falling into the lowest identity bins is always slightly higher than those of non-coding sequences (Dm-Dv: 49.22% of repeats vs 43.44% of non coding, Hs-Gg: 58.65% of repeats vs 40.21% of non coding). This tendency inverts around 65% identity for all species tested, and above that value, there is an excess of non-coding sequences as compared to repeats. In all species tested, we observed a shift of non-coding sequences towards high identity classes indicating that a fraction of non-coding sequences is under evolutionary constraints (Additional file 2A–B). The ratio for non-coding to coding sequences through different sequence identity classes is strikingly similar in vertebrates and insects (Additional file 2C–D). As in [14], we set a threshold for functional conservation at 80%. Table 2 shows the distribution of sequences above 80% identity through the different sequence classes. Repeats contribute marginally to these highly conserved sequences (0.52% in Hs-Gg alignments to 1.48% in Dm-Dv alignments). 47.69% of the sequences conserved in Dm-Dv, 58.79 % of these conserved in Hs-Gg are non-coding (Table 2). Our conservation threshold is more stringent that the one used by Siepel et al [5], so we report a higher proportion of coding bases within the conserved sequences (40.69% vs 28% for the vertebrates/and 50.83% vs 34% for the flies); however we are in agreement with the overall conclusion of their work, showing that a slightly higher proportion of non-coding bases are conserved in vertebrates than in insects.

**Table 2:** Functional distribution of sequences conserved with more than 80% identity

	***Hs-Gg***		***Dm-Dv***	
	***bp***	***%***	***bp***	***%***
**NC**	8'548'548	**58.79**	3'543'145	**47.69**
**REP**	75'140	**0.52**	110'029	**1.48**
**CDS**	5'917'357	**40.69**	3'776'986	**50.83**
	
**Total**	**14'541'045**		**7'430160**	

We then estimated the amount of conserved sequence information from the amount of conserved DNA falling into different percent identity classes. These estimates are based on a Markov model of mutations, similar to the Dayhoff model of protein evolution [15]. In essence, we assume that the apparent sequence identity *x *(where % identity is *x *× 100) observed in a sequence alignment depends on the mutational distance *d *between the compared sequences and the purifying selection coefficient *s *in the following manner:

x=r+(1−r)exp⁡(−(1−s)d1−r)
 MathType@MTEF@5@5@+=feaafiart1ev1aaatCvAUfKttLearuWrP9MDH5MBPbIqV92AaeXatLxBI9gBaebbnrfifHhDYfgasaacPC6xNi=xI8qiVKYPFjYdHaVhbbf9v8qqaqFr0xc9vqFj0dXdbba91qpepeI8k8fiI+fsY=rqGqVepae9pg0db9vqaiVgFr0xfr=xfr=xc9adbaqaaeGacaGaaiaabeqaaeqabiWaaaGcbaGaemiEaGNaeyypa0JaemOCaiNaey4kaSIaeiikaGIaeGymaeJaeyOeI0IaemOCaiNaeiykaKIagiyzauMaeiiEaGNaeiiCaa3aaeWaaeaacqGHsisldaWcaaqaaiabcIcaOiabigdaXiabgkHiTiabdohaZjabcMcaPiabdsgaKbqaaiabigdaXiabgkHiTiabdkhaYbaaaiaawIcacaGLPaaaaaa@463E@

Here, the parameter *r *is the equilibrium sequence identity value asymptotically reached after infinite divergence time, a parameter which can be empirically determined by aligning unrelated sequences from the same species pair. For gapped alignment algorithms, we typically find *r *values of approximately 0.45. The neutral distance *d *is the expected number of mutations per base-pair in the absence of any kind of selection. The estimates for *d *used in this work are derived from different sources (Additional file 1). Note that equation 1 corresponds to the Jukes-Cantor model [16] with a modified equilibrium value *r*.

For a given alignment, we define the amount of conserved sequence information as the number of bases in the reference sequence multiplied by the corresponding selection coefficient, which can be computed form the observed sequence identity by solving equation 1 for *s *(see Methods for more details). The resulting information is scaled in base-pair units (double bits). Following this principle, one can compute the total amount of sequence information conserved between two species from the number of bases contained in different conservation classes, as determined by our sliding window approach (see Methods). The amount of conserved sequence information is not identical to the amount of sequence information that is currently under selection in a given species. It is expected to decrease with increasing phenotypic divergence (although not much if one assumes that most biochemical and physiological processes are conserved within phyla).

**Figure 1:**
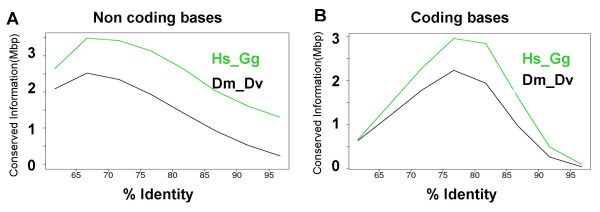
Distribution of conserved information in the coding and non-coding fractions of human and *Drosophila melanogaster *genomes, aligned respectively to chicken and *D. virilis *genomes. Total amount of conserved sequence information: Hs-Gg non-coding 17.6 Mb, coding 11.7 Mb, Dm-Dv non-coding 9.9 Mb, coding 8.5 Mb.

Figure 1 shows the distribution of conserved information for CDS and CNC in Human – chicken and *D. melanogaster *– D. *virilis *alignment *s*. The absolute amounts of conserved information are lower for the drosophilids alignments, both for coding and non-coding regions. However, the distributions of conserved information through different sequence identity classes are very similar for CDS in both species (Figure 1B). For non-coding sequences, the distributions of conserved information are clearly different from those for CDS, but also quite similar between the insect and vertebrate species pairs. A notable difference is nevertheless observed at the highest conservation level (above 90% identity), where vertebrates sequences greatly outnumber those from insects (1A, ~4 times more conserved information in the highest identity class). CNC account for 59.9% of conserved genomic information in vertebrates, and 53.9% in *D. melanogaster*-*D.virilis *alignments, which is very consistent with the values obtained by setting an arbitrary threshold at 80% identity (58.79% CNC in vertebrates and 47.69% CNC in drosophilids). The conservation of coding sequences in vertebrates and insects can be explained by general evolutionary constraints due to the requirements of protein translation and structure. The similarity of the distributions of non-coding conserved information suggests that the constraints causing the conservation of non-coding sequences in vertebrates and insects are likely to be as similar, and possibly as general as for the coding sequences.

### Vertebrate genomes are enriched in longer CNC

**Figure 2:**
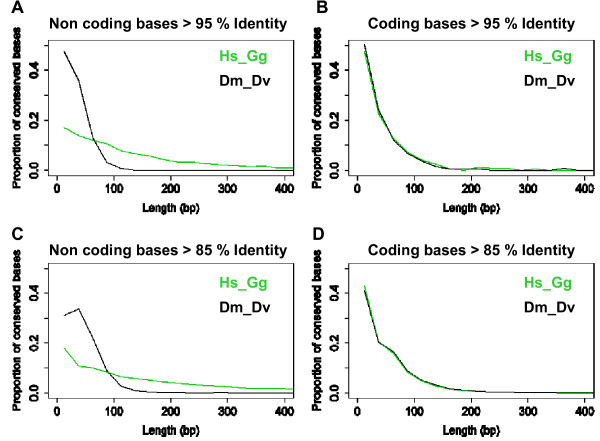
CNC persistence length. The figure shows the number of bases contained in conserved fragments of increasing length. In the fraction above 150 bp length and 85% identity (C and D), there are 2'097'887 non coding bases and 123'632 coding bases in Hs-Gg alignments, 12'864 noncoding bases and 84'381 coding bases in Dm-Dv alignments.

We established that the proportion of non-coding bases in the conserved information fraction is 1.09-fold lower in insect than in vertebrates (59.9% in vertebrates and 53.9% in insects), and the observed difference is unlikely to explain the apparent disparity in morphological complexity. We subsequently analyzed the persistence length of CNC in vertebrates and insects. We define the persistence length of a genomic region as the maximal length in which a threshold of percent identity level, measured in a sliding window, is maintained. To do this, we selected from pairwise alignments (Hs-Gg, Dm-Dv and Dm-Dp) all sequence windows where continuous sequence identity is >95%, >90% and >85%. The length distribution of conserved CDS in vertebrates and insects is similar. The length of insect CNC is very similar to coding sequences as well, but the CNC are clearly longer in vertebrates (Figure 2, Table 3). The length difference seems to be due to a heterogeneous set of longer fragments, and not to the presence of a large population of a defined length. An even more puzzling observation is that the average length of vertebrate CNC increases with sequence identity, (see Figure 2A and 2C), which strongly suggests a vertebrate specific role for the long, highly conserved elements.

**Table 3:** Composition of genomic regions of >95%, >90% and > 85% sequence identity in Hs-Gg and Dm-Dv alignments.

***Identity***	***Total***	***CNC***	***CDS***	***REP***	***%CDS***
**Hs-Gg > 95%**	**35235** *1449370*	**29714** *1246520*	**4224** *73889*	**76** *1048*	**11.99** *5.09*
**Hs-Gg > 90%**	**99150** *3721966*	**64310** *2766694*	**30223** *484097*	**213** *2286*	**30.48** *13.01*
**Hs-Gg > 85%**	**225366** *7875222*	**112328** *4706059*	**98632** *1839510*	**366** *3362*	**43.77** *23.36*
**Dm-Dv > 95%**	**16838** *299977*	**12626** *215714*	***2467*** 41230	**1299** *22143*	**14.65** *13.74*
**Dm-Dv > 90%**	**58427** *1164847*	**35038** *709230*	**18083** *294864*	**3556** *67176*	**30.95** *25.31*
**Dm-Dv > 85%**	**148049** *3336369*	**69424** *1599938*	**66419** *1297742*	**7536** *154698*	**44.86** *38.90*

For each identity class, we report the number of genomic fragments composed of (100%) coding, repetitive, or non coding regions, and (italics) encompassed base-pairs.

When we compare conservation in long regions (≥100 bp), a very clear difference appears between vertebrates and insects. In vertebrates, for a total number of 3794 ≥100 bp long, > 95% conserved fragments, we retrieve 3320 CNC, 411 partly coding and 63 coding (1,66 % CDS); in the Dm-Dv pair, we retrieve 86 fragments, including 20 CNC, 40 partly coding and 26 coding (30.23 % CDS). While the number of long, highly conserved CDS in both species is comparable, the number of partially coding, highly conserved sequences might reflect a higher conservation of UTR sequences in vertebrates. However, the 166-fold higher number of long CNC in vertebrates clearly suggests that they represent a sequence class absent in insects. If we consider regions of at least 150 bp, we find 1751 CNC in the human genome, and not a single one in the *D. melanogaster *genome, which is an even more striking difference.

To exclude the possibility that vertebrate long CNC are associated with known genes, we checked our set of vertebrate CNC for evidence of transcription. We extracted functional annotation from Ensembl, and classified the CNC as either a transcribed (and untranslated) part of an exon, or associated with a gene (within 1000 bp of an exon), or distant (> 1000 bp of an exon). In our set of 3794 non-coding fragments of a minimal length of 100 bp, 506 (13.34 %) are transcribed, 291 (7.76%) are associated with genes, and the remaining 2997 (79.0%) are located further than 1000 bp of any documented gene. This confirms previous reports that human-chicken conserved elements are often located far from genes [10]. It further suggests that most of these elements are not included in transcripts or proximal promoters of documented genes.

### Evolutionary dynamics

**Figure 3:**
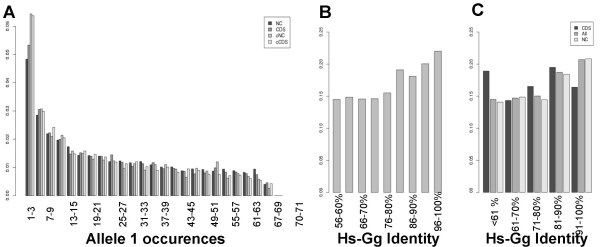
Population genetic analysis of human-chicken conserved sequences. Frequency spectra of non conserved coding (CDS) and non coding (NC) SNPs and of these conserved with more than 80% identity (cCDS and cNC), in bins of 3 alleles. B and C: Rare alleles (1 to 3 occurrences) frequency in different conservation classes, for all, coding or non coding sequences. The counts of rare and frequent alleles (>3 occurrences) were compared using the exact Fisher's test. The genotyped panel [17] includes 71 individuals (= 142 alleles).

It has previously been shown, based on HapMap SNP data, that vertebrate non-coding sequences are, indeed, selectively constrained [6]. The underlying evolutionary model predicts that the distribution of the less frequent alleles at polymorphic sites is shifted towards lower frequencies in regions subject to purifying selection. For each sequence and conservation class, we established the frequency spectra of human SNPs Figure 3A shows the allele frequency spectra for coding and non-coding, conserved (>80% identity) and non-conserved sequences, based on an unbiased genotyping of 71 individuals [17]. The conserved fraction of both coding and non-coding parts of the genome has a significantly lower proportion of rare alleles (1 to 3 occurrences) (p-value_coding _= 0.0138; p-value_noncoding _= 3.89e-11) confirming that it is selectively constrained. To investigate whether different sequence identity classes have different evolutionary constraints, we established the frequency spectra for each identity class (3B) or, to get a clearer view, for bins of 10% sequence identity (3C). Figures 3B and 3C show the frequency of rare alleles within each sequence identity class.

The overall frequency of rare alleles tends to increase with sequence identity (3B, 3C). which suggests that human non-coding sequences in distinct identity classes are subject to different evolutionary constraints. These results extend and complement the work of Drake et al [6]. We confirm, based on a separate, unbiased dataset, that CNC sequences are constrained, and that human-chicken conserved sequences, which evolved early in the vertebrate lineage have been maintained under selective pressure until our recent past.

**Figure 4:**
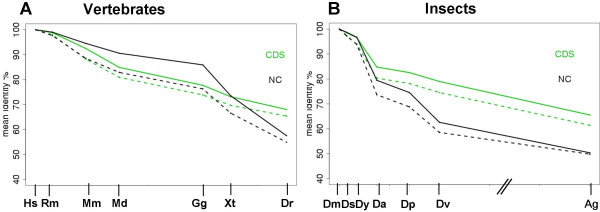
Persistence of coding and non-coding sequences in related species. A: Sequence identity % to human genome (B: Identity % to *D. melanogaster *genome) in whole genome alignments. CDS (green) > 80% protein coding regions; NC: 100% non-coding, non repetitive regions. Plain line: most conserved alignments, dotted line: 2nd fraction of conserved alignments. For each plot, the spacing is relative to the estimated geological divergence time (not to scale for *A. gambiae*) [13] (Species abbreviations are given in Additional file 1).

Yet vertebrate CNC are undetectable in insects [4], whereas most protein-coding regions maintain detectable sequence similarity between vertebrates and insects. Therefore we suspect that conserved protein-coding regions evolve under considerably more stable selection conditions than CNC. If our hypothesis is true, we should see different kinetic behavior of CNC and conserved coding regions in the vertebrate and insect lineages. To better understand this phenomenon, we systematically investigated the conservation of a large collection of coding and non-coding sequences through the evolutionary tree. We used as starting material UCSC multiple, whole genome vertebrate and insects alignments. The alignments were stratified based on sequence identity with closely related species, in 6 bins of equal size (see Methods). For the two most conserved alignment classes, we analyzed the persistence of the sequence similarity in more distantly related species. In vertebrates, the top bin of non-coding sequences is substantially more conserved than coding in all mammals and chicken (mean Hs-Gg identity 84.63% for non-coding alignments, and 70.76% for coding alignments). In more distant species, this ratio suddenly reverses, and in human-*Danio rerio *alignments, coding sequences are more conserved than non-coding (CNC: mean 38.58 % identity and CDS: 62.77%) (see Figure 4A). In the 2^nd ^class of conserved alignments (dotted lines), the conservation of non coding regions is very similar to CDS in closely related species, and, as for the first class, suddenly decreases in distant vertebrate species.

In drosophilids, CNC alignments are less conserved than CDS in most species, but the gap between coding and non-coding increases greatly in more distant species (see e.g. *D. virilis *and *A. gambiae*, Figure 4B). This result confirms that the *Drosophilids *genome has very few long highly conserved sequences. The rapid drop of the mean % identity of non coding alignments is due to the disappearance of CNC conservation in genome alignments of distant species, both for vertebrates and insects. For the most conserved class of vertebrate alignments, 58% of non coding regions are alignable between human and fish, versus 95% of coding alignments (66.48% identity in alignable non coding sequences, 69.23% in alignable CDS). These results clearly show that, in both vertebrates and insects, non-coding sequences have much shorter persistence time than coding sequences.

## Discussion

### Methodological choices

We analyzed the patterns of conservation of CNC in vertebrates and insects, and introduced for this purpose new methods and concepts. For most analyses, we used a sliding window technique which enables us to compute percent identity statistics for individual bases. This circumvents the problem of fragmenting genomes into conserved regions, a process that is very sensitive to the parameter settings of alignment algorithms and thus not robust. Moreover, there is no guarantee that conserved regions determined by computational procedures represent natural units of genome function and evolution. Our analysis of the persistence length of conserved sequence regions indicates that this approach is effective in unraveling conservation properties that are unique to vertebrate non-coding regions.

We chose to interpret genome-wide percent identity figures in the light of an evolutionary model enabling us to compute the total amount of conserved sequence information between two genomes. In doing so, we have opted for a simple but nevertheless flexible evolutionary model with few parameters. Flexibility results from an intrinsically simple way to take into account the effects of indels and biases due to the alignment scoring systems on observed % identity values. In fact, there is only one parameter that depends on the genome base composition and the alignment scoring system, the equilibrium identity value *r*, which can be readily determined by aligning large numbers of non-homologous sequences from the two genomes. The consistent results that we obtain for different species pairs indicate that our method of relating alignment -based sequence identity to conserved sequence information is an effective way to project % identity figures from species pairs with different mutational distances onto a comparable scale.

### CNC persistence length

One of the major results of our study is that the proportion of conserved non-coding to coding bases is similar in both taxa, as is the distribution of conserved information across different sequence identity classes. Yet we observe a very significant difference in the length distribution of these sequences: the majority of conserved vertebrate base pairs occur in conserved DNA fragments longer than 100 bp, whereas insects CNC are organized in short fragments, closely mirroring the length distribution of conserved coding sequences. Our set of extremely long CNC reflect the same phenomena as vertebrate ultra-conserved elements (UCE) [3]. UCE are defined as sequence segments longer than 200 base-pairs which are absolutely conserved between human, mouse and rat. Like persistently long sequence regions unraveled by our approach, they are unique to vertebrates and mostly non-coding. However, altogether they represent only about 120'000 bp. Conversely, the fraction of bases contained in persistently conserved sequence regions of at least 85% identity and length 150 or longer totals 2.1 million bases. At the same time, this fraction is about 20 times enriched in non-coding bases (94.4%). Our results indicate that UCE are thus just the tip of the iceberg of a much larger class of vertebrate-specific non-coding regions with unique conservation properties.

What is the function of these long CNC? Several publications focusing on a subset of highly conserved elements suggest that some of them are distant regulators of developmental genes [4,8,18,19]. A large-scale proof of the enhancer activity of these sequences has recently been produced [9]. Based on the increasing amount of recent evidence, we postulate that most of the highly conserved CNC are distal regulators of gene expression. Likewise, conserved elements in drosophilids and worms were found to occur in the vicinity of developmental regulators and transcription factors [20]. Interestingly, and consistent with our results, drosophilid cis-regulatory elements have been postulated to be typically less than 50 bp [11]. If so, the striking CNC length differences suggest that conserved regulatory elements are much longer in vertebrates than insects, which indicates that more transcription factor binding sites might be included in a regulatory module, and suggests a more complex regulation of gene expression in vertebrates.

### CNC persistence time

To understand the rules governing the evolution of CNC, we investigated the persistence time of a large set of coding and non-coding sequences through the evolutionary trees of insects and vertebrates. In closely related vertebrate species, we observe that CNC are more conserved than coding regions. However, the reversal of this trend at larger evolutionary distances shows that CNC have a shorter persistence time than coding regions. A similar evolutionary phenomenon occurred at least twice, in vertebrates and in insects, where we observe a relative slow-down of coding region evolution over longer time periods. In the light of these observations, the complete disappearance of perceptible sequence conservation of non-coding regions across phyla appears to be the continuation of a trend that is also operating within phylum, though perhaps at lower intensity.

To our knowledge, this is the first systematic report describing the kinetic aspects of the evolution of non-coding regions of a whole genome. A couple of previous studies addressed this question for specific non-coding regions. Consistent with our observation, UCE as defined by Bejerano and coworkers [3] were found to have accelerated evolutionary rates in the lineages leading to birds and amphibians, as compared to the rates observed between mammals. A study on CNC evolution in the Hox gene cluster shows that the evolution of these regions is significantly faster in *Xenopus*, compared to more closely related mammalian species. The authors postulate a faster evolution of cis-regulatory sequences in the amphibian lineage, as well as in the stem lineage of mammals [21].

### Evolutionary bases of CNC persistence time

In order to understand the evolutionary forces that lead to different kinetic behaviors of CNC and coding regions, one has to compare the observations to predictions made by various models. The classical Dayhoff model upon which our estimation of conserved sequence information is based, assumes gene-specific evolutionary rates due to varying degrees of purifying selection. The overall strength of selection remains constant over time for a given gene; however, the specific constraints acting on a particular base will change, allowing for any possible base substitution over sufficiently long time periods. According to the Dayhoff model, orthologous sequences evolving in two different lineages will asymptotically approach the saturation percent identity value characteristic of alignments of unrelated sequences (formula 1).

The kinetic behavior of conserved coding regions as shown in Fig. 4 is not compatible with this model. The relatively high divergence observed between closely related species probably reflects rapid saturation with silent mutations and conservative amino acid replacements. The slower rate documented by more distant time points is consistent with the assumption that a fraction of the coding regions code for proteins that retain the same function throughout the animal kingdom. This fraction presumably evolves under invariable functional constraints that prohibit complete sequence divergence.

The evolutionary kinetics of insect CNC could in principle be explained by a Dayhoff process as described by formula 1. On the other hand, the time-course of vertebrate CNC shows accelerated evolutionary rates in the lineages leading to amphibians and fishes, which is not compatible with a neutral model of molecular evolution and suggest that specific evolutionary constraints underlie the divergence patterns of these sequences.

### CNC persistence time and current view of body plan evolution

This conservation dynamics of vertebrate CNC is perhaps best explained by an evolutionary process consisting of long periods of high stability alternating with short periods of rapid and pervasive adaptive changes. In fact, such a scenario is part of an emerging view of animal body plan evolution put forward, for instance, in recent publications by Davidson, Prud'homme, and others [22-24]. The key assumptions underlying their model are: (i) different body plans of insects, vertebrates, worms and other phyla are executed by the same "toolbox" of genes, many of them encoding transcription factors, (ii) the different body plans result from different temporal and spatial expression patterns of toolbox genes, (iii) the different expression patterns result from changes in the *cis-*regulatory regions of individual genes, not from changes in the sequence specificity of the cognate *trans-*acting transcription factors. According to this view, morphological changes are the result of a rewiring of a hierarchical gene regulatory network via *cis-*regulatory mutations. Conversely, body plan stability requires high conservation of the *cis-*regulatory regions of toolbox genes.

This model is supported by several studies indicating that many of the highly conserved CNC may in fact be tissue-specific enhancers of developmental genes (see for example[9]). Particularly relevant in this context is the work by Prabhakar et al. showing that non-coding regions conserved between primates, but lacking visible conservation in more distant vertebrates are active transcriptional regulators [25]. The idea of a common toolbox is based on the intriguing observation that phyla-specific CNC of insects, worms and vertebrates, are associated with overlapping sets of conserved developmental genes [24]. Several studies proved that changes in CNC produce a morphological phenotype via a change in the expression pattern of a nearby gene. For example, the different expression pattern of Hoxc8 in the thoracic region of mouse and chicken is associated with a relative expansion of the cervical region in the chicken. This crucial difference in the expression of a developmental gene is caused by a conserved enhancer region, differing by only a few nucleotides between mouse and chicken. The chicken Hoxc8 enhancer is sufficient to reproduce a chicken-like expression pattern in the mouse [26]. Several observations on insect wing color and other developmental patterns, as well as the pelvic reduction in stickleback fishes (reviewed by[23]) further suggest that morphological evolution often occurs via cis-regulatory changes that affect the expression of broadly conserved genes.

In view of the above model, the specific conservation kinetics of vertebrate CNC suggests a partial rewiring of developmental gene regulatory network at the early stages of amniote evolution. At later stages, the low rate of CNC changes speaks for high stability of major parts of body plan in lineages leading to chicken and man. Note further that differential mutation rates cannot explain the slower evolution of CNC relative to coding regions, since the level of Hs-Gg conservation strongly correlates with the selective constraints revealed by data from human population studies. Thus, the most stringent subset of highly conserved CNC has most likely evolved in amniotes and has been exposed to a constantly high selective pressure from the human-chicken common ancestor up to the recent spread-out of the human populations. This set of sequences is thus likely to contain key regions regulating amniote development.

## Conclusion

Here we introduced the concepts of persistence time and length, and applied them to characterize evolutionary kinetics of coding and non-coding regions. We analyzed the length distribution of CNC in vertebrates and insects. Our results show that a similar proportion of conserved coding to non coding regions exists in vertebrates and insects, but they are organized in longer fragments in vertebrates. This observation gives insight into the design principles of regulatory regions in both phyla. We also show that non coding sequences have a much shorter evolutionary persistence time than coding sequences. Our results might explain why vertebrate CNC are not found in other phyla, and suggest that non-coding regions are an important factor of morphological evolution. With more genomes becoming available, more detailed analyses based on these criteria will help associate individual CNC with lineage specific physiological and morphological changes.

## Methods

### Sliding window method for measuring local sequence conservation

The following pairwise Blastz genomic alignments were downloaded from the UCSC FTP repository [27]: Dm-Dv: dm2(BGDPv.4) – droVir1, Hs-Gg: hg18-galGal3 [28-30] (see Additional file 1 for species abbreviations). Sequence identity percentage was computed in an asymmetric fashion for the reference species (Hs or Dm) by counting the number of conserved bases in a sliding window containing 60 bp of the reference species. This number is used to compute the percent identity for the base at the center of the window (pos. 30). By moving the window in 1 bp steps, this procedure yields a % identity value for each aligned base of the reference genome in an aligned region. Bases closer than 30 bp to the limits of aligned regions are assigned the values of the first or last window, respectively.

The sequence identities were assigned to 10 discrete bins. The first includes the aligned sequence with 55 % or less identity (33 of 60 bases), and the next bins increase in 5% increments until 100% identity (last bin: 58–60 conserved identical bases). Each base in the reference genomes was further classified as coding or non-coding based on Ensembl genome annotation [31] (drosophila melanogaster_core_37_4e, and *homo sapiens *core_45_36g). Perl scripts based on the Ensembl Perl API [32] were used for this purpose.

### Quantitative assessment of conserved information

Selection coefficients were computed from the median sequence identity of each bin (e.g. 0.983 for most conserved class) by solving equation (1) in the Results Section. The equilibrium identity value *r *was determined empirically for each species pair in the following way: 900 pairs of genomic segments of length 60 were extracted randomly from the genomes and aligned with the program align (Myers & Miller, CABIOS 4:11–17) from the fasta2 package [33]. For each alignment, the identity level was computed as the fraction of bases in the reference sequence that are paired with identical bases in the target sequence. Note that align reports a different type % identity value, obtained by dividing the number of identical residue pairs by the length of the alignment including gaps. The alignments were generated with the HoxD55.q matrix downloaded from the UCSC genome browser web site. This matrix also provides default gap penalties which we left unchanged. The mean % identity for random sequences obtained in this way was 44.1% for Dm-Dv and 42.8% for Hs-Gg.

The total amount of conserved information for a species pair was obtained by summing the conserved information for percent identity classes which correspond to a selection coefficient between 0 and 1 according to equation 1, i.e. the classes with sequence identity equal or higher than value corresponding to the neutral mutational distance of the two species (parameter *d*).

### SNP data analysis

Selective pressure for CNC was computed based on Perlegen allele frequency data [17]. The data, obtained from the authors for the human genome version hg16, were 'lifted' to the hg18 version using the liftOver program) and corresponding chain files downloaded from the UCSC web site [34].

From 61'746 known SNPs in the human-chicken alignable regions, we used 41'775 in this study, for which genotyping data were available for all 71 individuals of the Perlegen panel. Each genotyped SNP was annotated with the Ensembl annotation. The sequence identity percentage, and allele frequency spectra were established for the different functional and sequence identity classes tested. Rare (1–3 alleles in 71 individuals) and frequent (>3) alleles' counts were compared using the exact Fisher's test.

### Persistence time analysis

To establish the persistence time of CNC, we used whole genome multiple alignments downloaded from the UCSC genome browser (17-species vertebrate alignments and 8-species insect alignments). Vertebrate genomes used in this analysis included hg17, rheMac2, mm7, monDom2, galGal2, xenTro1 and danRer3. Insect alignments included dm2, droSim1, droYak1, droAna1, dp3, droVir1 and anoGam1. We selected a subset of alignments at least 100 bp long, and including at least 6 of 7 of our target species. Identity percentage with reference genomes hg17 or dm2 was calculated for all species as described before. Identity was set to mutational equilibrium values (44% for insects and 43% vertebrates) for target regions missing in the alignments or including >50% of gaps.

The selected set included 44'709 insect alignments and 181'556 vertebrate alignments, with an average length of 120 bp. For each alignment, the bases of the reference genome sequence were labeled as coding, non-coding or repeat based on Ensembl annotations. We define as CDS all sequences with at least 80% of coding bases, and as NC, all sequences consisting exclusively of non-coding bases (excluding repeats). Alignments not satisfying either of these criteria were excluded. We split the set of alignments in 6 bins of equal size, stratifying them by the mean % identity values observed with two close target species. For vertebrates, we considered macaque and mouse; for insects *D. simulans *and *D. yakuba*. The two most conserved bins of the four classes (vertebrate/insect, coding/non-coding) were considered for further analysis.

## Authors' contributions

DR and PB conceived the study, DR, EB, CN and CVJ performed the data analysis, DR, CVJ and PB wrote the manuscript. All authors read and approved the manuscript.

## Supplementary Material

Additional file 1 Genomic distances. Unconstrained genomic distances relative to *Homo sapiens (Hs) *and *Drosophila melanogaster (Dm) *in vertebrates and insects. Click here for file

Additional file 2 Distribution of conserved bases. Distribution of coding sequence, repeats, and non coding non repetitive regions over the different sequence identity classes. Click here for file
