# Supplementary material for: Vertebrate conserved non coding DNA regions have a high persistence length and a short persistence time
Source: BMC Genomics. 2007 Oct 31;8:398. doi: 10.1186/1471-2164-8-398 (PMC2211324; doi:10.1186/1471-2164-8-398)
Supplement: Additional file 1 — Genomic distances. Unconstrained genomic distances relative to Homo sapiens (Hs) and Drosophila melanogaster (Dm) in vertebrates and insects. [file 1471-2164-8-398-S1.doc]

|  | Substitutions per synonymous site | Genomic distance (d)) | Evolutionary time (MYA) |
| --- | --- | --- | --- |
| ***Hs –Rm*** *(Macaca mulatta)* | 0.052* | ***0.076*** | 30 |
| ***Hs – Mm*** *(Mus musculus)* | 0.420* | 0.620 *** | 90 |
| ***Hs – Md*** *(Monodelphis domestica)* | 0.600* | ***0.886*** | 150 |
| ***Hs – Gg*** *(Gallus gallus)* | 0.930* | ***1.372*** | 300 |
| ***Hs – Xt*** *(Xenopus tropicalis)* | - | ***1.6*** | 350 |
| ***Hs – Dr*** *(Danio rerio)* | 1.916* | ***2.828*** | 450 |
| ***Dm – Ds*** *(Drosophila simulans)* | - | ***0.118*** | 5.4** |
| ***Dm – Dy*** *(Drosophila yakuba)* | - | ***0.284*** | 12.8** |
| ***Dm – Da*** *(Drosophila ananassae)* | - | ***0.982*** | 44.2** |
| ***Dm – Dp*** *(Drosophila pseudoobscura)* | - | ***1.218*** | 54.9** |
| ***Dm – Dv*** *(Drosophila virilis)* | - | ***1.396*** | 62.9** |
| ***Dm – Ag*** *(Anopheles gambiae)* | - | ***5.55*** | 250 |

Additional file 1: Unconstrained genomic distances relative to *Homo sapiens (Hs)* and *Drosophila melanogaster (Dm)* in vertebrates and insects. This information was collected from the following sources *[1]; **[2]; ***[3]. The values in bold were computed from the available data as follows: for vertebrates, *d*=synonymous sites/0.677; for *Drosophila*, *d*=MYA*11.1*2/1000.

**Additional References:**

1. EH Margulies, VV Maduro, PJ Thomas, JP Tomkins, CT Amemiya, M Luo, ED Green: **Comparative sequencing provides insights about the structure and conservation of marsupial and monotreme genomes**. *Proc Natl Acad Sci U S A* 2005, **102**:3354-9.

2. K Tamura, S Subramanian, S Kumar: **Temporal patterns of fruit fly (Drosophila) evolution revealed by mutation clocks**. *Mol Biol Evol* 2004, **21**:36-44.

3. GM Cooper, M Brudno, ED Green, S Batzoglou, A Sidow: **Quantitative estimates of sequence divergence for comparative analyses of mammalian genomes**. *Genome Res* 2003, **13**:813-20.
