# Supplementary material for: Vertebrate conserved non coding DNA regions have a high persistence length and a short persistence time
Source: BMC Genomics. 2007 Oct 31;8:398. doi: 10.1186/1471-2164-8-398 (PMC2211324; doi:10.1186/1471-2164-8-398)
Supplement: Additional file 2 — Distribution of conserved bases. Distribution of coding sequence, repeats, and non coding non repetitive regions over the different sequence identity classes. [file 1471-2164-8-398-S2.ppt]

## Slide 1
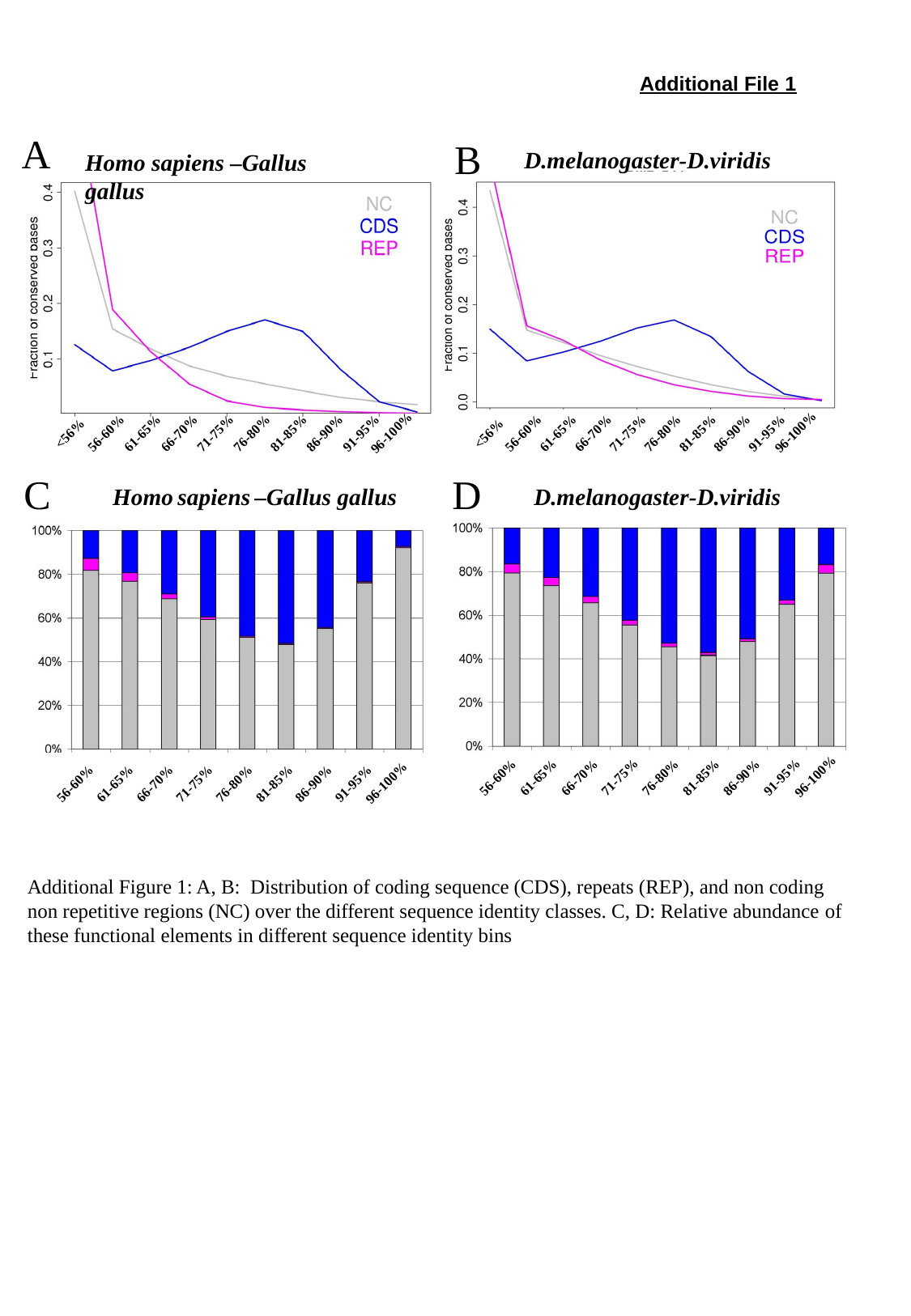

Additional File 1
A
B
D.melanogaster-D.viridis
Homo sapiens –Gallus gallus
96-100%
<56%
56-60%
61-65%
66-70%
71-75%
76-80%
81-85%
86-90%
91-95%
96-100%
<56%
56-60%
61-65%
66-70%
71-75%
76-80%
81-85%
86-90%
91-95%
C
D
Homo sapiens –Gallus gallus
D.melanogaster-D.viridis
96-100%
56-60%
61-65%
66-70%
71-75%
76-80%
81-85%
86-90%
91-95%
96-100%
56-60%
61-65%
66-70%
71-75%
76-80%
81-85%
86-90%
91-95%
Additional Figure 1: A, B: Distribution of coding sequence (CDS), repeats (REP), and non coding non repetitive regions (NC) over the different sequence identity classes. C, D: Relative abundance of these functional elements in different sequence identity bins
